# Notch signalling regulates asymmetric division and inter-conversion between lgr5 and bmi1 expressing intestinal stem cells

**DOI:** 10.1038/srep26069

**Published:** 2016-05-16

**Authors:** Tara Srinivasan, Elaine Bich Than, Pengcheng Bu, Kuei-Ling Tung, Kai-Yuan Chen, Leonard Augenlicht, Steven M. Lipkin, Xiling Shen

**Affiliations:** 1 Department of Biomedical Engineering, Cornell University, Ithaca, New York, 14853, USA; 2 Departments of Medicine, Surgery and Pathology, Weill Cornell Medical College, New York City, New York, 10021, USA; 3 School of Electrical and Computer Engineering, Cornell University, Ithaca, New York, 14853, USA; 4 Department of Biological and Environmental Engineering, Cornell University, Ithaca, New York, 14853, USA; 5 Department of Oncology, Montefiore Medical Center, Albert Einstein College of Medicine, Bronx, NY, 10461 USA; 6 Department of Biomedical Engineering, Duke University, Durham, North Carolina, 27708 USA

## Abstract

Rapidly cycling LGR5+ intestinal stem cells (ISCs) located at the base of crypts are the primary driver of regeneration. Additionally, BMI1 expression is correlated with a slow cycling pool of ISCs located at +4 position. While previous reports have shown interconversion between these two populations following tissue injury, we provide evidence that NOTCH signaling regulates the balance between these two populations and promotes asymmetric division as a mechanism for interconversion in the mouse intestine. In both *in vitro* and *in vivo* models, NOTCH suppression reduces the ratio of BMI1+/LGR5+ ISCs while NOTCH stimulation increases this ratio. Furthermore, NOTCH signaling can activate asymmetric division after intestinal inflammation. Overall, these data provide insights into ISC plasticity, demonstrating a direct interconversion mechanism between slow- and fast-cycling ISCs.

In the murine intestine^1^, fast-cycling LGR5-expressing (Leucine-rich repeat-containing G protein-coupled receptor 5-expressing) crypt base columnar (CBC) stem cells are the cells primarily responsible for maintaining homeostasis by replacing cells as they mature and are sloughed into the lumen. LGR5+ CBCs can self-renew, or produce transit-amplifying (TA) daughter cells that rapidly divide and terminally differentiate into distinct lineages that populate the intestinal epithelium^1^,^2^. There are also additional stem or progenitor cell populations^3^, which have been associated with markers including BMI1, HOPX, TERT and LRIG-1^4^,^5^,^6^,^7^,^8^. Single-molecule transcript analyses suggest that the presence of LGR5 and BMI1 mRNAs is more prevalent than that indicated by antibody staining and that they potentially overlap in a subset of cells, raising the possibility that post-translational mechanisms may amplify the difference in protein levels and these two populations may be more plastic than previously thought^9^,^10^.

Remarkably, it has been shown that Lgr5+ stem cells can produce +4 cells as daughters^4^, and +4 ISCs can reciprocally produce Lgr5+ CBC daughter cells as a compensatory mechanism following experimental ablation of Lgr5-expressing cells^4^,^8^. The interconversion between faster proliferating Lgr5+ vs. more quiescent Bmi1+ ISC populations demonstrates the fluidity of crypt cell type hierarchy, which can help maintain homeostasis and adapt to different types of intestinal micro-environmental conditions. Our newly gained knowledge about ISC plasticity provokes the question as to which mechanism regulates the choice of each identity.

In mouse intestinal crypts, Notch signaling is known to be an important pathway associated with stem cell self-renewal^2^,^11^,^12^,^13^,^14^. Accordingly, the proliferative zone of intestinal crypts contains essential Notch pathway components, such as receptors NOTCH1 and NOTCH2, ligands DLL-1, DLL-4, and JAG-1, and downstream effector genes Hairy and Enhancer of Split 1 (Hes1) and Hes5^14^,^15^,^16^. Here we demonstrate that NOTCH signaling is a key mechanism that regulates the balance between highly proliferative and relatively quiescent stem cells, and activates asymmetric division when the tissue is under stress. Maintaining both fast- and slow-cycling stem cells may provide a survival strategy for maintaining homeostasis within intestinal tissue.

## Results

### NOTCH signaling balances BMI1+ and LGR5+ populations in intestinal organoids

**Figure 1:**
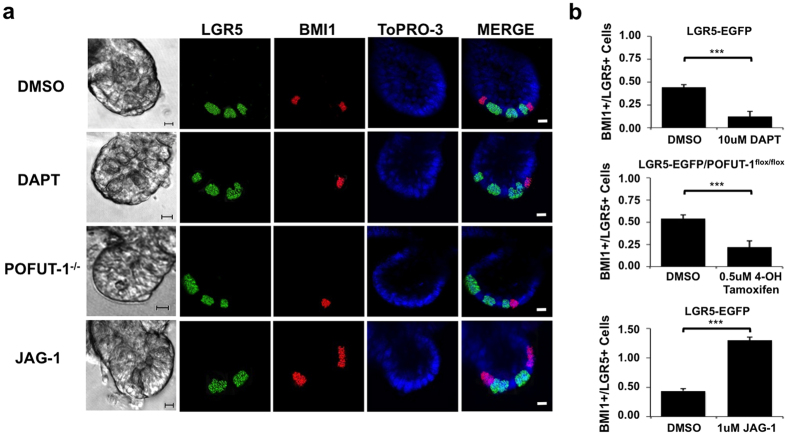
NOTCH regulates the balance between LGR5+ and BMI1+ ISC populations. (**a**) Mouse LGR5-EGFP or LGR5-EGFP-creER/POFUT-1^**flox/flox**^ ISCs (propagated as organoids) after 48-hour treatment with DMSO (Control), 10 uM DAPT, 0.5 uM 4-OH-Tamoxifen to induce POFUT-1^−/−^ phenotype, or 1 uM JAG-1. Anti-GFP antibody (green) detects LGR5-GFP+ cells; BMI1 (red) and ToPRO-3 (blue) labels nuclei. Scale bar represents 20 μm. (**b**) Quantification of BMI1+ and LGR5+ cells for conditions in (**a**). Data is shown as mean ± s.d of three independent experiments with n = 500 organoids/replicate measured (***p = 0.001; Student t-test).

Single mouse LGR5-EGFP+ intestinal stem cells (ISCs) were isolated (Supplementary Fig. 1A) using FACS^17^ and propagated as organoids to quantify the relative ratio of BMI1+ and LGR5+ ISC under conditions in which NOTCH signaling was modulated^17^. When NOTCH signaling was inhibited with the γ-secretase inhibitor DAPT for 48 hours and visualized by co-IF, the ratio of BMI1+/LGR5+ cells decreased vs. DMSO-treated controls (p = 0.001; Student t-test) (Fig. 1a,b). Western analysis for NICD confirmed inhibition of NOTCH activity due to DAPT treatment (Supplementary Fig. 1B).

POFUT-1 (Protein *O*-fucosyltransferase 1) is an enzyme responsible for the addition of fucose by O-linkage on EGF domains of NOTCH receptors and is required for functional NOTCH signaling^18^,^19^,^20^. To confirm the results from chemical inhibition of NOTCH signaling, ISCs derived from mice expressing a LGR5-EGFP-creER/POFUT-1^flox/flox^ genotype were treated *in vitro* with 4-hydroxy-Tamoxifen for approximately 48 hours to inactivate the POFUT-1 gene, and cells were then imaged by IF (Fig. 1a). Similar to DAPT treatment, the BMI1+/LGR5+ cell ratio decreased vs. DMSO-treated controls (p = 0.001; Student t-test) (Fig. 1b). Western analysis showed, as expected, that POFUT-1 and NICD were not detectable in this model of NOTCH suppression (Supplementary Fig. 1C).

A complementary experiment then examined the effect of stimulation of the NOTCH pathway via soluble JAG-1 embedded in Matrigel, the substrate on which the organoids were propagated^4^. JAG-1 stimulation of NOTCH in ISCs generated from LGR5-EGFP mice significantly increased the ratio of BMI1+/LGR5+ ISCs vs. DMSO-treated controls (p = 0.001; Student t-test) (Fig. 1a,b). As expected, JAG-1 treatment also increased NICD levels (Supplementary Fig. 1D).

The effects on NOTCH modulation on intestinal stem cell populations *in vitro* were then further validated based on ASCL2 expression, an alternative marker for LGR5+ ISCs^12^,^21^. Consistent with our earlier findings, DAPT treatment and POFUT-1 deletion decreased the ratio of BMI1+/ASCL2+ ISCs while JAG-1 stimulation increased the ratio of BMI1+/ASCL2+ compared to DMSO-treated controls (p = 0.001; Student t-test) (Supplementary Fig. 1E,F). Taken together, these findings show that NOTCH signaling increases the ratio of BMI1+/LGR5+ (ASCL2+) ISCs, whereas NOTCH inhibition reduces this ratio in intestinal organoids.

### NOTCH signaling balances BMI1+ and LGR5+ populations *in vivo*

**Figure 2:**
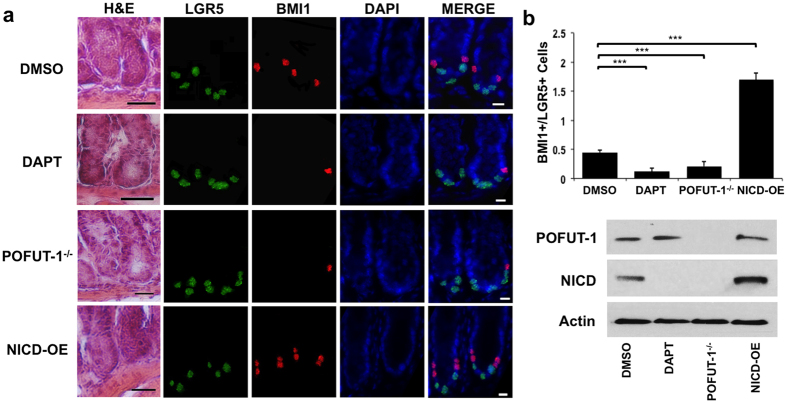
NOTCH balances LGR5+ and BMI1+ ISC populations *in vivo*. (**a**) Treatments were administered by i.p injections: DMSO (on LGR5-EGFP mice); DAPT (on LGR5-EGFP mice every 12 hours for 3 days); Tamoxifen (on LGR5-EGFP-creER/POFUT-1^flox/flox^ mice every 24 hours for 5 consecutive days); or Tamoxifen (on LGR5-EGFP-CreERT2/Rosa26-YFP-NICD mice every 24 hours for 8 consecutive days). Shown are representative intestinal crypts from the duodenum: Anti-GFP antibody (green) detects LGR5 (green); BMI1 (red) and DAPI (blue). Scale bar: 200 μm (H&E), 20 μm (IF). (**b**) Top: Quantification of BMI1+ and LGR5+ cells (n = 5 mice/treatment) for conditions in (**a**). Data represents mean ± s.d of 5 mice/condition with n = 500 crypts/mouse measured (***p = 0.001, one-way ANOVA). Bottom: Western Blot for POFUT-1 and NICD expression. Actin was used as a control.

To confirm the organoid studies, intestinal sections from LGR5-EGFP mice treated with DMSO were analyzed by IF for LGR5 (detected by GFP antibody) and BMI1 expression. BMI1+ cells largely localized to nuclei in the +4 position and LGR5+ cells were found interspersed at the crypt base (Fig. 2a). As previously reported, antibody staining of LGR5-GFP and BMI1 is more specific than single-molecule RNA FISH, suggesting that post-translational mechanisms may amplify the difference in protein levels^10^. LGR5-EGFP mice were then treated with DAPT by i.p injections to inhibit NOTCH signaling. Quantification over multiple crypts showed a marked reduction in the ratio of BMI1+/LGR5+ ISCs compared to the DMSO-treated control group (p = 0.001; one-way ANOVA) (Fig. 2a,b). NOTCH signaling was also inhibited using LGR5-EGFP-creER/POFUT-1^flox/flox^ mice that were administered daily Tamoxifen i.p injections to ablate POFUT-1. Similar to DAPT treatment, the ratio of BMI1+/LGR5+ ISCs was significantly reduced in the POFUT-1^−/−^ vs. DMSO-treated control group (p = 0.001; one-way ANOVA (Fig. 2a,b). Protein expression analysis confirmed POFUT-1 was not detected in POFUT-1^−/−^ tissue. Similarly, western blotting for NICD expression from protein of intestinal tissue confirmed NOTCH suppression by DAPT and POFUT-1^−/−^ (Fig. 2b). We also analyzed intestinal crypts of POFUT-1^flox/flox^:Villin-cre mice^18^, which have constitutive deletion of POFUT-1 driven by the Villin promoter (Supplementary Fig. 2A). Similar reduced ratios of BMI1+/LGR5+ (ASCL2+) were observed in both inducible and constitutive POFUT-1 knockout mouse models.

Finally, a Rosa26-YFP-NICD mouse strain was crossed with the LGR5-EGFP-CreERT2 strain to generate LGR5-EGFP-CreERT2, Rosa26-YFP-NICD mice to model NOTCH stimulation via NICD expression in ISC following Tamoxifen induction. Co-IF based on LGR5 and BMI1 expression showed elevated BMI1 staining in cells located around the +4 position while LGR5 expression was similar to the DMSO-treated control group (Fig. 2a). The ratio of the number of ISCs expressing BMI1 relative to LGR5 was significantly increased compared to the control (p = 0.001; one-way ANOVA (Fig. 2b). Western blot analysis confirmed NICD overexpression from harvested intestinal tissue (Fig. 2b). Quantification for each *in vivo* condition was confirmed based on ASCL2 expression (Supplementary Fig. 2B,C), suggesting that NOTCH suppression decreases the ratio of BMI1+/LGR5 + (ASCL2+) ISCs while NOTCH overexpression elevates this ratio. Overall, our observation of BMI1+ cells *in vitro* and *in vivo* is consistent with a role for NOTCH signaling to drive production of BMI1+ ISC (Supplementary Fig. 3A,B).

### Asymmetric BMI1+/LGR5+ division of ISC organoid cells

**Figure 3:**
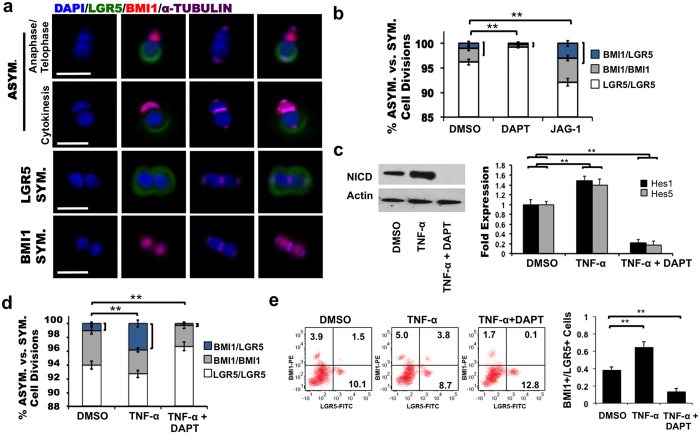
NOTCH promotes asymmetric BMI1+/LGR5+ ISC division. (**a**) LGR5-EGFP ISC daughter pair just prior to completion of asymmetric (top) or LGR5+ symmetric (middle) or BMI1+ symmetric (bottom) cell division. LGR5 (green), BMI1 (red), α-TUBULIN (purple), DAPI (blue). Scale bar: 50 μm. (**b**) LGR5-EGFP ISCs were treated with DMSO, 10 uM DAPT or 1 uM JAG-1 for 16 h in a pair cell assay. Shown is percentage of ISCs undergoing BMI1+/LGR5+ asymmetric (blue), BMI1+/BMI1+  symmetric (grey), or LGR5+/LGR5+ symmetric (white) cell division determined by co-IF for LGR5, BMI1, and α-TUBULIN expression (**p = 0.002, one-way ANOVA). Data represents mean ± s.d. from three independent experiments with n = 500 TUBULIN+ pairs/replicate measured. (**c**) Single LGR5-EGFP ISCs were treated with DMSO, 10 ng/ml TNF-α, or both 10 ng/ml TNF-α and 10 uM DAPT. Shown is Western blot analysis for NICD expression (left) and RT-PCR data of NOTCH effector genes Hes1 and Hes5 (right) under each experimental condition (performed in triplicate and presented mean ± s.d.; p = 0.01; Student t-test for statistical significance). (**d**) LGR5-EGFP ISCs were treated with 10 ng/ml TNF-α for 72 hours with simultaneous administration of DMSO or 10 uM DAPT during the last 48 hours. Shown is percentage of ISCs undergoing BMI1+/LGR5+ asymmetric (blue), BMI1+/BMI1+ symmetric (grey), or LGR5+/LGR5+ symmetric (white) cell division (**p = 0.003 (TNF-α), p = 0.002 (TNF-α+DAPT); one-way ANOVA). Data represents mean ± s.d. from three independent experiments with n = 500 TUBULIN+ pairs/replicate measured. (**e**) Representative FACS plot (left) and quantitative analysis based on FACS data (right) for each condition in (d). Data is shown as mean ± s.d of three independent experiments with n = 1 × 10^6^ cells/replicate measured (**p = 0.01; Student t-test).

To assess a potential role for NOTCH signaling in regulating LGR5+ and BMI1+ (HOPX+) normal stem cell populations, we examined *in vitro* organoid cultures of single ISCs derived from mice carrying an EGFP knock-in driven by the LGR5 promoter (LGR5-EGFP). Murine crypts were isolated, dissociated into single cells, embedded in Matrigel overlaid with growth media, and observed 16 hours post-plating to visualize the mitotic outcome of single stem cells by IF. Using α-TUBULIN staining, we observed single ISCs producing BMI1+/LGR5+, LGR5+/LGR5+, and BMI1+/BMI1+ daughter pairs in the final stages of cell division (Fig. 3a). To confirm antibody specificity, ISCs were treated with a microtubule-depolymerizing agent (Colchicine) for 4 hours following the pair cell assay, which showed an absence of α-TUBULIN expression in Ki67+ dividing pairs (Supplementary Fig. 4A). Next, we tested additional microtubule markers, including β-TUBULIN (Supplementary Fig. 4B) and γ-TUBULIN (Supplementary Fig. 4C) in pair cell assays, which consistently showed the generation of BMI1+/LGR5+ asymmetric ISC daughters. We also found asymmetric distribution of LGR5 and the cell polarity marker PARD3A in ISC daughter pairs prior to completion of cell division using mitotic spindle labeling, indicating intrinsic asymmetric division (Supplementary Fig. 4D).

Single ISCs were analyzed further using the pair cell assay for conditions that modulate NOTCH signaling (Fig. 3b). RT-PCR analysis of Hes1 and Hes5 confirmed NOTCH decreased and increased signaling upon treatment with DAPT or JAG-1, respectively (Supplementary Fig. 4E). The frequency of BMI1+/LGR5+ cell pairs was reduced upon NOTCH inhibition and increased upon NOTCH stimulation relative to the DMSO-treated control (p = 0.002; one-way ANOVA). Quantification based on ASCL2 expression was consistent with these findings, indicating that DAPT decreased the percentage of asymmetric BMI1+/ASCL2+ division while JAG-1 elevated this frequency compared to DMSO-treated controls (Supplementary Fig. 5A,B). To understand whether this process can be influenced by stress to the system, we treated organoids with TNF-α, a pro-inflammatory cytokine linked to chronic colitis and carcinogenesis^22^,^23^ and increased apoptosis of organoid cells^24^. TNF-α was administered at a low dosage of 10ng/ml to LGR5-EGFP ISCs over 72 hours. We found that TNF-α up-regulated NICD by Western blot analysis, as well as expression of Hes1 and Hes5 by RT-PCR (Fig. 3c). TNF-α treated ISCs showed a marked increase to 4.3% BMI1+/LGR5+ divisions (p = 0.003, one-way ANOVA) (Fig. 3d). When DAPT was added to the culture medium during the last 48 hours of TNF-α treatment, NICD, Hes1 and Hes5 levels all decreased (Fig. 3c) and notably, BMI1+/LGR5+ asymmetric division was reduced to 0.1% (p = 0.002, one-way ANOVA) (Fig. 3d). Quantification based on ASCL2 expression was consistent with these results, indicating that TNF-α increased the percentage of asymmetric BMI1+/ASCL2+ division while TNF-α + DAPT decreased this frequency compared to DMSO-treated controls (Supplementary Fig. 5C). FACS analysis was then used to quantify the BMI1+ vs. LGR5+ ISC population balance in TNF-α, and TNF-α+DAPT treatment groups (Fig. 3e). Consistent with our earlier findings, the BMI1+/LGR5+ double positive population containing BMI1+/LGR+ pairs increased with TNF-α treatment and decreased with TNF-α + DAPT treatment. The ratio of BMI1+/LGR5+ ISCs increased with TNF-α treatment and decreased with TNF-α + DAPT treatment (p = 0.01; Student t-test). These data suggest that normal ISCs are capable of NOTCH-dependent asymmetric BMI1+/LGR5+ division, which can be triggered by stress.

### Asymmetric BMI1+/LGR5+ division *in vivo*

**Figure 4:**
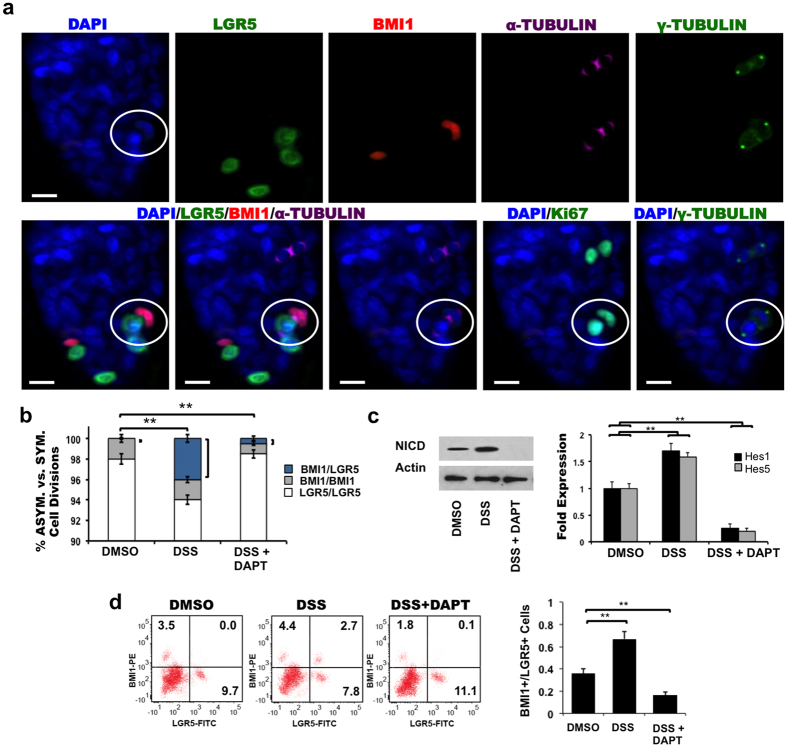
NOTCH regulates BMI1+/LGR5+ ISC division. (**a**) LGR5-EGFP mice were administered 3% DSS in the drinking water for 5 days, followed by plain water for 5 days. During the last three days of the plain water diet, mice were injected i.p. with DMSO or DAPT every 12 hours. Shown are duodenal intestinal crypts: LGR5 (green), BMI1 (red), α-TUBULIN (purple), Ki67 (green), γ-TUBULIN (green). Scale bar: 20 μm. (**b**) Quantitative analysis from mice (n = 5/treatment) for conditions in (**a**). Shown is the frequency of BMI1+/LGR5+ asymmetric (blue), BMI1+/BMI1+ symmetric (grey), or LGR5+/LGR5+ symmetric (white) cell division. Data represents mean ± s.d. of 5 mice/condition with n = 500 TUBULIN+ dividing pairs/mouse measured (**p = 0.002 (DSS), p = 0.004 (DSS+DAPT); one-way ANOVA). (**c**) *In vivo* assays were performed as described in (**a**). Shown is corresponding western blot analysis for NICD expression (left) and RT-PCR data of NOTCH effector genes Hes1 and Hes5 (right) under each experimental condition (performed in triplicate and presented mean ± s.d; **p = 0.01; Student t-test for statistical significance). (**d**) Representative FACS plot (left) and quantitative analysis based on FACS data (right) for each condition in (**a**). Data is shown as mean ± s.d of 5 mice/condition with n = 5 × 10^6^ cells/mouse measured (**p = 0.01; Student t-test).

LGR5-EGFP intestinal tissue was then used to study ISC division using α-TUBULIN and Ki67 expression. We detected only LGR5+/LGR5+ and BMI1+/BMI1+ symmetric division (Supplementary Fig. 6A). This finding shows that asymmetric BMI1+/LGR5+ division under homeostatic conditions *in vivo* is rare, unlike in organoids, where stem cells are promoted to proliferate by growth factors such as WNT, R-SPONDIN, and NOGGIN. To examine the effect of stress on ISC division, LGR5-EGFP mice were treated with 3% dextran sodium sulfate (DSS) in the drinking water for 5 days followed by a 5-day recovery period with plain water^23^. DSS has been shown to promote small intestinal inflammation^25^,^26^,^27^ in addition to chronic colonic inflammation that increases intestinal cell apoptosis. Since BMI1+ ISCs are not present in the colon^3^,^8^, the effects of DSS on BMI1+/LGR5+ asymmetric division were evaluated in the small intestine. Consistent with TNF-α treatment in organoids, we detected asymmetric BMI1+/LGR5+ daughters in α-TUBULIN+/γ-TUBULIN+/Ki67+ dividing pairs (Fig. 4a). DSS treatment increased BMI1+/LGR5+ asymmetric division frequency to 3.9% (p = 0.002; one-way ANOVA) (Fig. 4b). When DSS-treated mice were injected with DAPT during the last 3 days of the plain water diet, the number of asymmetric BMI1+/LGR5+ cell pairs reduced to 0.2% (p = 0.004; one-way ANOVA) (Fig. 4b). Quantification based on ASCL2 expression was consistent with these results, indicating that DSS increased the percentage of asymmetric BMI1+/ASCL2+ division while DSS + DAPT decreased this frequency compared to the control (Supplementary Fig. 6B,C). DSS treatment increased NOTCH signaling levels, while the addition of DAPT reduced NOTCH signaling levels, in terms of NICD, Hes1, and Hes5 expression (Fig. 4c). DSS and DSS + DAPT intestinal tissues were then analyzed by FACS to quantify BMI1+ vs. LGR5+ population balance (Fig. 4d). Again, the double-positive population containing BMI1+/LGR5+ pairs increased with DSS treatment and decreased with DSS + DAPT treatment. The ratio of BMI1+/LGR5+ ISCs increased with DSS treatment and decreased with DSS + DAPT treatment (p = 0.01; Student t-test). Therefore, these data suggest that stress can trigger asymmetric BMI1+/LGR5+ division in the intestine, potentially increasing conversion between BMI1+ and LGR5+ cells.

## Discussion

We show that BMI1+/LGR5+ divisions, regulated by NOTCH signaling levels, exist in mouse intestinal organoids and in the intestinal mucosa. In mouse intestine, LGR5+ CBCs are fast-cycling and proliferate largely through symmetric division^2^,^28^, while BMI1+/HOPX+ cells are mostly quiescent. However, single-molecule RNA FISH suggests that the mRNA levels of these markers do not as clearly distinguish the fast and slow cycling populations, raising the possibility of plasticity and interconversion among these populations^9^,^10^. Targeted ablation of LGR5+ ISCs in transgenic mice with diphtheria toxin revealed that intestinal crypt homeostasis could be rescued by rare, normally quiescent ISCs^8^. Remarkably, the two populations can be replenished when each is depleted^4^. Potential plasticity between LGR5+ ISCs and other quiescent cell types in response to tissue injury has also been suggested^29^.

Our data suggest potential roles for the NOTCH pathway to regulate the balance between fast- and slow-cycling populations, and asymmetric BMI1+/LGR5+ division can potentially be activated to aid direct interconversion when the balance is disrupted and needs to be restored. The frequency of such BMI1+/LGR5 division does not need to be high, given that the normally quiescent ISCs are relatively rare and long-lasting. However, the low frequency of quiescent cells may still serve an important role as reserve stem cells, establishing an important link to repopulation and maintenance of homeostasis.

## Methods

### Animal Experiments

LGR5-EGFP (also known as Lgr5-EGFP-IRES-creERT2) mice originally purchased from the Jackson Laboratory and LGR5-EGFP-creER/POFUT-1^flox/flox^ mice on a mixed 129/C57BL/6 background were provided by Dr. Augenlicht’s research group. For *in vivo* studies, DAPT was administered every 12 hours for 3 days by i.p injection in LGR5-EGFP mice, and Tamoxifen (Sigma) was administered by daily i.p injections for 5 consecutive days in POFUT-1^flox/flox^ mice. For DSS treatment, LGR5-EGFP mice were administered 3% Dextran Sodium Sulfate (DSS) (MP Biomedicals) in the drinking water for 5 days, followed by plain water for 5 days. During the last three days of the plain water diet, mice were injected i.p. with DAPT according to the regimen described earlier. The entire length of the small intestine was harvested for RT-PCR and Western blotting analyses or snap frozen in O.C.T, cryo-sectioned, and stained by IF. Additionally, harvested single intestinal cells were subjected to FACS analysis using a Beckman Coulter flow cytometer to detect LGR5-EGFP and BMI1 expression. FlowJo software was used to analyze data and to gate populations according to 7-AAD viability, and forward and side scattering. Cutoff thresholds were provided by using unstained cells as a negative control. All experiments were performed in accordance with the ethical and care guidelines established by the Research Animal Resource Center of Weill Cornell Medical College followed the protocol (2009-0029). Additionally, all experimental protocols were approved by the Research Animal Resource Center of Weill Cornell Medical College.

### Murine ISC Analysis

LGR5-EGFP+ ISCs from LGR5-EGFP and LGR5-EGFP-creER/POFUT-1^flox/flox^ mice were isolated using FACS analysis and cultured as organoids as previously described^17^. For *in vitro* studies, LGR5-EGFP organoids were seeded on chamber slides and treated with one of the following: 10uM DAPT (EMD Millipore) added to the media for 48 hours^30^, or embedded in Matrigel containing 1uM JAG-1 (AnaSpec) for 48 hours^4^ followed by IF. LGR5-EGFP-creER/POFUT-1^flox/flox^ ISCs were treated with 500nM 4-hydroxytamoxifen (Sigma) added to the media for 48 hours to induce Cre recombinase followed by IF. Single ISCs were embedded in Matrigel overlaid with growth medium and incubated at 37 °C for 16 hours before IF in pair-cell assays.

### Pair-Cell Assay

Pair-cell analysis was performed as described^31^. For pair-cell assays involving single mouse intestinal stem cells (ISCs), cells were embedded in Matrigel overlaid with growth medium and incubated at 37 °C and 5% CO_2_ for 16 hours before fixation and IF. Specifically, single LGR5-EGFP ISCs were treated with one of the following: DMSO, 10 uM DAPT (EMD Millipore) for 48 hours^30^, or 1 uM JAG-1 (AnaSpec) for 48 hours^4^. ISCs were then fixed and stained for BMI1, LGR5 and TUBULIN expression to observe dividing pairs. In order to determine TUBULIN antibody specificity following a 16 hour pair cell assay, single ISCs were treated with 10 μM colchicine (Santa Cruz: cat # 64-86-8) for 4 hours prior to fixation^32^. In a separate pair cell assay, single LGR5-EGFP ISCs were treated with 10 ng/ml TNF-α (R&D) dissolved in culture medium for 72 hours. TNF-α–treated ISCs were simultaneously treated with DMSO or DAPT during the last 48 hours as described above. Subsequently, ISCs were subjected to FACS analysis for BMI1 and LGR5 expression.

### Statistical Analysis

The data displayed are represented as mean ± s.d. Statistical comparisons between two groups was made using Student t-test or one-way ANOVA for multiple groups. *P* < 0.05 was used to establish statistical significance.

## Additional Information

**How to cite this article**: Srinivasan, T. *et al*. Notch signalling regulates asymmetric division and inter-conversion between lgr5 and bmi1 expressing intestinal stem cells. *Sci. Rep*. **6**, 26069; doi: 10.1038/srep26069 (2016).

## Supplementary Material

Supplementary Information
