# Supplementary material for: Notch signalling regulates asymmetric division and inter-conversion between lgr5 and bmi1 expressing intestinal stem cells
Source: Sci Rep. 2016 May 16;6:26069. doi: 10.1038/srep26069 (PMC4867651; doi:10.1038/srep26069)
Supplement: Supplementary Information [file srep26069-s1.pdf]

NOTCH Signaling Regulates Fast- and Slow-Cycling Intestinal Stem Cells

Tara Srinivasan[1], Elaine Bich Than[3], Pengcheng Bu[1,2], Kuei-Ling Tung[4], Kai-Yuan Chen[1], Leonard Augenlicht[5], Steven M. Lipkin[3], and Xiling Shen[1,2,6]

[1]Department of Biomedical Engineering, Cornell University. [2]School of Electrical and Computer Engineering, Cornell University. [3]Departments of Medicine, Surgery and Pathology, Weill Cornell Medical College. [4]Department of Biological and Environmental Engineering, Cornell University. [5]Department of Oncology, Montefiore Medical Center, Albert Einstein College of Medicine. [6]Department of Biomedical Engineering, Duke University.

SUPPLEMENTAL METHODS

Antibodies

**Primary Antibody**	**Supplier**	**Catalog Number**	**Dilution***
Anti-ASCL2	Bioss	bs-12349R	1:100 (IF)
Anti-α-TUBULIN	Abcam	Ab6160	1:500 (IF)
Anti-β-TUBULIN	Sigma Aldrich	T8328	1:100 (IF)
Anti-γ-TUBULIN	Abcam	Ab11316	1:100 (IF)
anti-β-ACTIN	Abcam	ab6276	1:4000 (WB)
Anti-BMI1 (for IF murine specificity)	Cell Signaling	5856	1:50 (IF)
Anti-BMI1 (for FACS murine specificity)	Abcam	Ab14389	1:100 (FACS)
anti-GFP	Abcam	ab5450	1:100 (IF)
anti-NICD	Cell Signaling	2421	1:1000 (WB)
anti-PARD3A	Santa Cruz	sc-79577	1:100 (IF)
anti-POFUT1	Abcam	ab154051	1:2000 (WB)

*Application: IF: (Immunofluorescence); WB: (Western Blotting)

The corresponding Alexa Fluor® -488 or Alexa Fluor® -555 conjugated secondary antibodies were purchased from Life Technologies. Alexa Fluor® 647 secondary antibody (anti-rat: A-21472; anti-mouse: A-21463; anti-rabbit: A-21443) was purchased from Life Technologies.

Animal experiments for Mouse Intestinal Stem Cell Studies

LGR5-EGFP and LGR5-EGFP-creER/POFUT-1$^{flox/flox}$ mice on a mixed 129/C57BL/6 background were a generous gift from Dr. Leonard Augenlicht's laboratory. Mice were genotyped to confirm allelic identity using PCR analysis with LGR5 common forward primer: 5'- CTGCTCTCTGCTCCCAGTCT-3', LGR5 WT reverse primer: 5'-ATACCCCATCCCTTTTGAGC-3', LGR5 mutant reverse primer: 5'-GAACTTCAGGGTCAGCTTGC -3', POFUT-1 forward primer: 5'-GGGTCACCTTCATGTACAAGTGAGTG-3' and POFUT-1 reverse primer: 5'-

ACCCACAGGCTGTGCAGTCTTTG-3'. The thermocyling profile used for PCR amplification is described as follows: for LGR5-EGFP: 94°C (3 min)/[94°C (30 sec), 66°C (30 sec), 72°C (30 sec)] for 35 cycles/72°C (2 min) and for POFUT-1: 94°C (2 min)/[94°C (30 sec), 58°C (30 sec), 72°C (90 sec)] for 34 cycles/72°C (5 min). Daily intraperitoneal (i.p) injections of Tamoxifen (Sigma) at a dose of 75 mg/kg dissolved in sterile corn oil were used for 5 consecutive days to induce Cre enzyme activity in 8 week old POFUT-1$^{flox/flox}$ mice [1]. The DAPT treatment regimen for LGR5-EGFP/POFUT-1$^{+/+}$ mice was 200 mg/kg every 12 hours for 3 days delivered by i.p injection [1]. As a control, mice were treated with DMSO (Sigma) at the corresponding time points. For DSS treatment, 8 week old LGR5-EGFP mice were administered 3% Dextran Sodium Sulfate (DSS) (36,000–50,000 kDa; MP Biomedicals) in the drinking water for 5 days, followed by plain water for 5 days. During the last three days of the plain water diet, mice were injected i.p. with DMSO or DAPT according to the regimen described earlier. All experiments were performed in accordance with the ethical and care guidelines established by the Research Animal Resource Center of Weill Cornell Medical College followed the protocol (2009-0029).

Intestinal Crypt isolation and Organoid Culture

The entire length of the small intestines and colons were harvested and opened longitudinally immediately after sacrificing 8-week-old untreated LGR5-EGFP and LGR5-EGFP-creER/POFUT-1$^{flox/flox}$ mice for establishing organoid culture. Villi were removed gently using a glass coverslip. Murine tissue was washed with cold phosphate-buffered saline (PBS), cut into 5.0 mm fragments and again washed with cold PBS. Tissue fragments were incubated in 2.0mM EDTA in PBS on ice for 30 minutes followed by replacement of the EDTA medium with cold PBS. To release the villous fraction, a 10mL pipette was used to vigorously resuspend the tissue several times and the supernatant was discarded. The remaining tissue was then suspended in cold PBS and mechanically agitated several times to release crypts into the supernatant. Crypt

fractions were collected and centrifuged at 6,000 RPM for 5 minutes to determine which fractions were most enriched by microscopic examination. The appropriate fractions were combined, diluted in cold PBS, and centrifuged to yield a crypt-containing pellet. The pellet was then diluted in Advanced DMEM/F12 (Invitrogen) containing Glutamax (Invitrogen), passed through 70um filter and centrifuged again to purify crypts for organoid culture following single cell dissociation. In order to achieve single cell dissociation from crypts, purified crypt pellets were incubated at 37°C in SMEM (Invitrogen) that contained 0.8 KU/ml DNase (Sigma), 10 uM ROCK pathway inhibitor Y-27632 (Sigma), and 1 mg/mL Trypsin-EDTA (Invitrogen) for 30 minutes. Single cells were then passed though a 40um filter and subjected to FACS analysis to isolate LGR5-EGFP+ intestinal stem cells (ISCs) for organoid culture. LGR5-GFP expression was detected by FITC emission. FACS was conducted using a Beckman Coulter flow cytometer. FlowJo software was used to analyze data and to gate populations according to 7-AAD viability, and forward and side scattering. Cutoff thresholds were provided by using unstained cells as a negative control.

Single LGR5-EGFP+ ISCs were embedded in 50ul Matrigel (BD Biosciences) seeded on pre-warmed 24-well plates at a concentration of 1000 cells or crypts/mL. Following Matrigel polymerization, complete ISC media was added to each well. The formulation for ISC media is as follows: Advanced DMEM/F12 (Invitrogen) supplemented with Glutamax (Invitrogen), 10mM HEPES (Invitogen), N2 (Invitrogen), B27 without vitamin A (Invitrogen), and 1 uM *N*-acetylcysteine (Sigma). To ensure bioactivity of growth factors, a separate solution ISC media containing 50ng/mL EGF (Invitrogen), 100ng/mL Noggin (Peprotech), and 10% R-SPONDIN1 conditioned media (generated in house) was freshly prepared each passage. Growth factors were added every 2 days with full media replacement every 4 days. Passage occurred once per week at

a ratio of 1:4 by removing organoids from Matrigel with cold PBS, mechanically disrupted using a fire-polished glass pipette and resuspended in fresh Matrigel.

***In vitro* Mouse Intestinal Stem Cell Studies**

For *in vitro* studies, LGR5-EGFP organoids were seeded on chamber slides and treated with one of the following: 10uM DAPT (EMD Millipore) added to the media for 48 hours [2], or embedded in Matrigel containing 1uM JAG-1 (AnaSpec) for 48 hours [3]. LGR5-EGFP-creER/POFUT-1$^{flox/flox}$ ISCs were treated with 500nM 4-hydroxytamoxifen (Sigma) added to the media for 48 hours to induce Cre recombinase and generate LGR5-EGFP-creER/POFUT-1$^{-/-}$ ISCs [4]. For FACS analysis, harvested organoids were mechanically dissociated and resuspended in 2 mls PBS and passed through a 40um filter to collect single cells. After cell counting, the suspension was incubated with 7-AAD dye to assess viability and a BMI1 primary antibody that was subsequently labeled with PE. LGR5-GFP expression was detected by FITC emission. FlowJo software was used to analyze data and to gate populations according to 7-AAD viability, and forward and side scattering. Cutoff thresholds were provided by using unstained cells as a negative control.

Immunofluorescence of Murine Crypts or *in vitro* organoids

The entire length of intestinal tissues of LGR5-EGFP mice and LGR5-EGFP-creER/POFUT-1$^{flox/flox}$ mice treated *in vivo* as described earlier (n=5 per condition) were harvested, snap frozen in O.C.T, and cryo-sectioned. Frozen intestinal tissue from 8-week-old POFUT-1$^{flox/flox}$:Villin-cre mice [5] were a gift from Dr. Augenlicht's laboratory. Briefly, POFUT-1$^{flox/flox}$:Villin-cre mice were previously generated by crossing POFUT-1$^{lox/flox}$ mice on a mixed 129/C57BL/6 background with a constitutive Tg(Vil-cre)997Gum line in which Cre recombinase expression was driven by the villin promoter. Frozen intestinal tissue corresponding to NICD overexpression (OE) was a generous gift from Xiling Shen's laboratory. Briefly,

LGR5-EGFP-CreERT2 x Rosa26-YFP-NICD knock-in mouse strain was treated daily with 75 mg/kg Tamoxifen for 8 consecutive days to induce Cre enzyme activity and NICD-OE phenotype. All murine frozen tissue specimens were fixed with 4% PFA for co-immunofluorescence using BMI1, LGR5 (detected by GFP), or ASCL2 expression according to the protocol described below. DAPI (Invitrogen) was used as a counterstain for IF on the inverted fluorescent microscope. Murine frozen tissue sections were also used for staining with Hematoxylin and Eosin according to standard protocol. LGR5 and BMI1 expression was quantified within individual crypts of n = 5 mice/condition with n=500 crypts/mouse measured.

Intestinal organoids embedded in Matrigel that were treated under stated conditions were either fixed with 4% PFA for 15 minutes at room temperature for co-immunofluorescence according to the established protocol below or harvested for protein analysis via Western Blotting using methods previously described [6]. Visualization of organoids was performed using BMI1 and LGR5 (detected by GFP) expression and ToPro-3 as a nuclear counterstain on a Zeiss LSM 510 laser scanning confocal microscope using an Apo 40× 1.40 oil objective. Images were collected and analyzed with confocal software (LSM 510 Meta; Zeiss). The experiment was performed in triplicate and LGR5 and BMI1 expression was quantified within individual organoids using n=500 organoids/replicate.

Immunofluorescence

Following fixation, cells were permeabilized with 0.2% Triton X-100 and incubated in a blocking solution (5% BSA or normal serum (goat, rabbit or horse) and 0.1% Triton-X in PBS) for 1 hour. For single or co-immunofluorescence staining, primary antibodies diluted in blocking solution were added overnight at 4°C overnight. To ensure specificity, a no primary antibody control staining was performed. The slides were then washed in PBS and incubated with the appropriate secondary antibody for 1 hour at room temperature and counterstained/mounted with

Vectashield containing DAPI (Vector Laboratories). For ASCL2 staining, antigen retrieval was performed as previously described [7] and incorporated into the protocol for IF.

Quantitative RT-PCR and Protein Analysis

A Qiagen RNeasy Plus kit was used to extract total RNA from crypts over the entire length of the small intestine. This was followed by reverse transcription to cDNA using ABI Taqman Reverse Transcription kit (Applied Biosystems). ABI Taqman Master mix and ABI Prism HT7900 were used to run quantitative real-time PCR. Taqman primers (ABI) purchased from Life Technologies were used for HES1 (Product ID: Mm01342805_m1), HES5 (Product ID: Mm00439311_g1), and GAPDH (control) (Product ID: Mm99999915_g1). The data represents the average result of three independent experiments normalized to GAPDH with error bars designating S.E.M. Protein isolation from ISCs and harvested mouse intestinal tissue as well as methods for Western Blotting were performed as previously described [6]. β-actin was used as a control for normalization.

REFERENCES

1. Breunig, J.J., et al., *Notch regulates cell fate and dendrite morphology of newborn neurons in the postnatal dentate gyrus.* Proc Natl Acad Sci U S A, 2007. **104**(51): p. 20558-63.
2. Sikandar, S.S., et al., *NOTCH signaling is required for formation and self-renewal of tumor-initiating cells and for repression of secretory cell differentiation in colon cancer.* Cancer Res, 2010. **70**(4): p. 1469-78.
3. Takeda, N., et al., *Interconversion between intestinal stem cell populations in distinct niches.* Science, 2011. **334**(6061): p. 1420-4.
4. Wiener, Z., et al., *Oncogenic mutations in intestinal adenomas regulate Bim-mediated apoptosis induced by TGF-beta.* Proc Natl Acad Sci U S A, 2014. **111**(21): p. E2229-36.
5. Guilmeau, S., et al., *Intestinal deletion of Pofut1 in the mouse inactivates notch signaling and causes enterocolitis.* Gastroenterology, 2008. **135**(3): p. 849-60, 860 e1-6.
6. Pan, Z., et al., *Impaired placental trophoblast lineage differentiation in Alkbh1(-/-) mice.* Dev Dyn, 2008. **237**(2): p. 316-27.
7. Yamashita, S. and Y. Okada, *Application of heat-induced antigen retrieval to aldehyde-fixed fresh frozen sections.* J Histochem Cytochem, 2005. **53**(11): p. 1421-32.

Supplementary Fig. 1

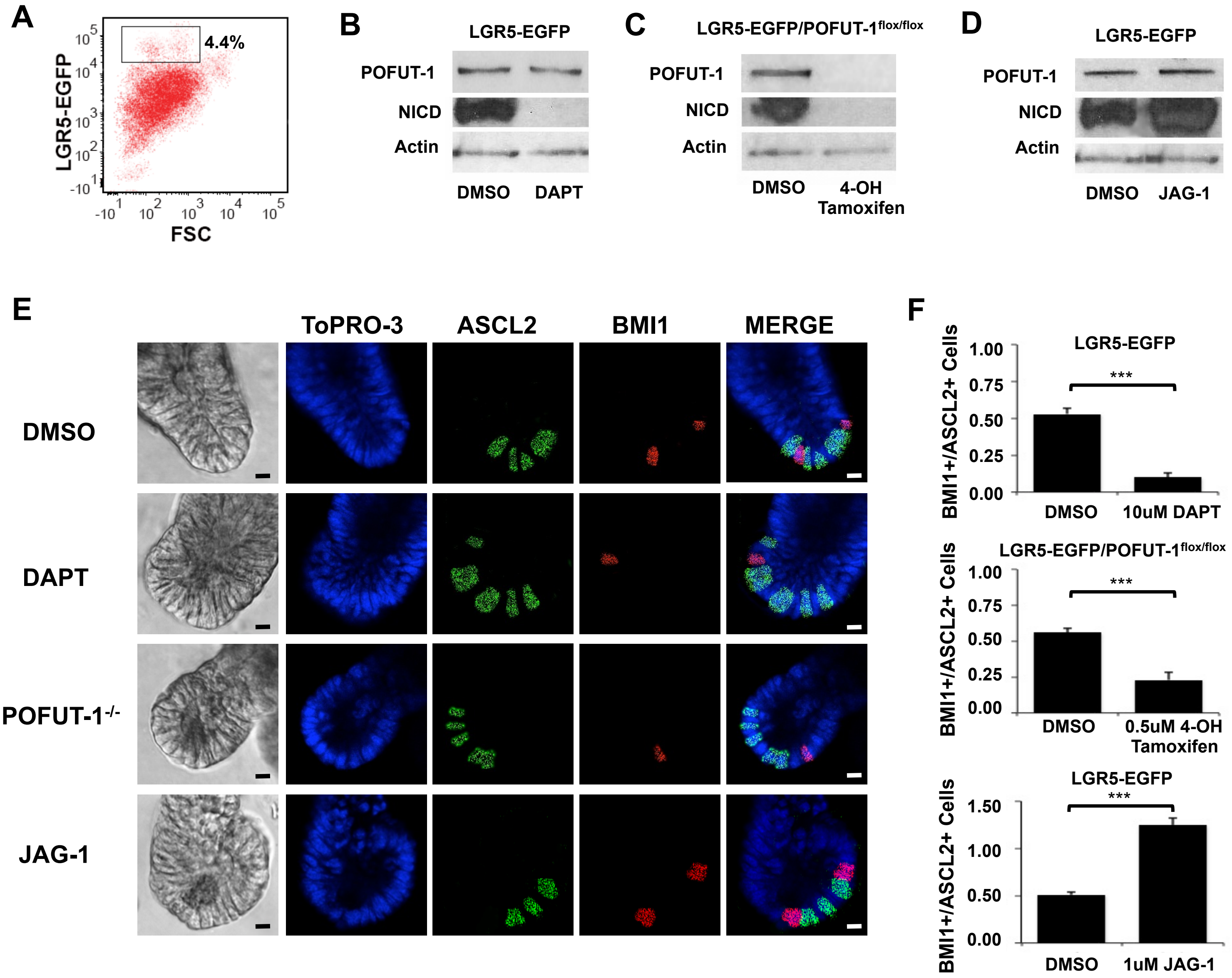

Supplementary Fig. 1. Analysis of murine ISC organoids under NOTCH modulation conditions. Related to Fig. 1.

(**A**) Representative FACS plot of harvested intestinal cells from LGR5-EGFP mice, including gated analysis to isolate LGR5-EGFP+ intestinal stem cells (ISCs) for subsequent organoid culture. (**B**) Western blot data from LGR5-EGFP ISC organoids, demonstrating suppression of NICD by DAPT treatment. Actin was used as a loading control. (**C**) Western blot data from LGR5-EGFP-creER/POFUT-1$^{\mathbf{flox/flox}}$ ISC organoids following 4-OH Tamoxifen induction of POFUT-1$^{-/-}$ phenotype, showing an absence of POFUT-1 and NICD. Actin was used as a loading control. (**D**) Western blot data from LGR5-EGFP ISC organoids after JAG-1 treatment indicating increased NICD expression. Actin was used as a loading control. (**E**) Mouse LGR5-EGFP or LGR5-EGFP-creER/POFUT-1$^{\mathbf{flox/flox}}$ ISCs (propagated as organoids) after 48-hour treatment with DMSO (Control), 10uM DAPT, 0.5uM 4-OH-Tamoxifen to induce POFUT-1$^{-/-}$ phenotype, or 1uM JAG-1. ASCL2 (green) detects ISCs; BMI1 (red) and ToPRO-3 (blue) labels nuclei. Scale bar represents 20μm. (**F**) Quantification of BMI1+ and ASCL2+ cells for conditions in (E). Data represents mean ± s.d. of three independent experiments with n=500 organoids/replicate measured (***, p=0.001; Student t-test).

Supplementary Fig. 2

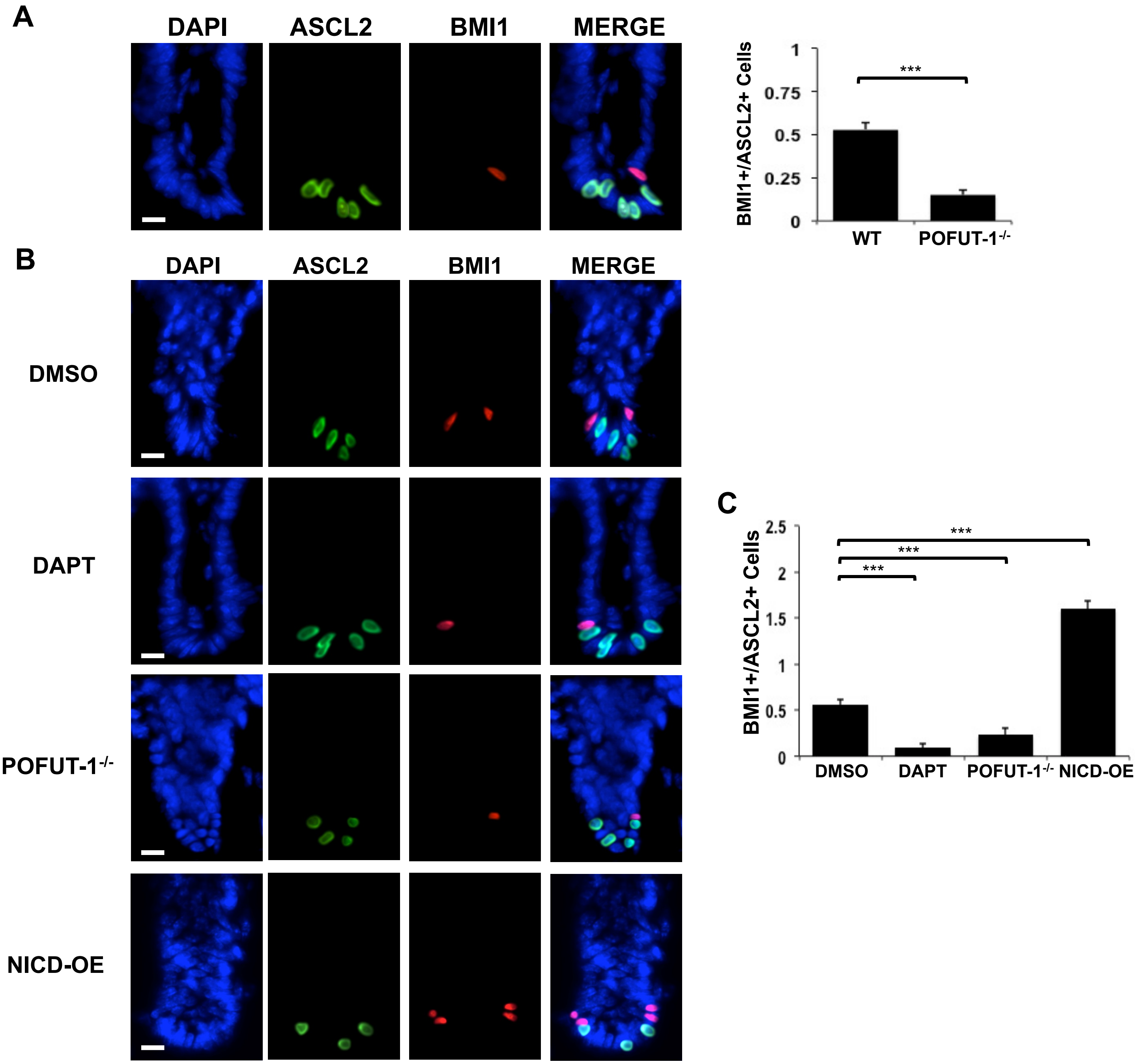

Supplementary Fig. 2. NOTCH modulation of ISC populations *in vivo*. Related to Fig. 2.

(**A**) POFUT-1$^{flox/flox}$:Villin-cre mice with constitutive deletion of POFUT-1 driven by the Villin promoter. Left: Shown is a representative intestinal crypt in the duodenum with co-immunoflourescence: ASCL2 (green); BMI1 (red) and DAPI (blue). Scale bar: 20μm. Right: Quantification of BMI1+ and ASCL2+ cells. Data represents mean ± s.d of 5 mice with n=500 crypts per mouse measured (***, p=0.001, one-way ANOVA). **(B)** Treatments were administered by i.p injections: DMSO (on LGR5-EGFP mice); DAPT (on LGR5-EGFP mice every 12 hours for 3 days); Tamoxifen (on LGR5-EGFP-creER/POFUT-1$^{flox/flox}$ mice every 24 hours for 5 consecutive days); or Tamoxifen (on LGR5-EGFP-CreERT2/Rosa26-YFP-NICD mice every 24 hours for 8 consecutive days). Shown are representative intestinal crypts from the duodenum: ASCL2 (green); BMI1 (red) and DAPI (blue). Scale bar: 20μm. (**C**) Quantification of BMI1+ and ASCL2+ cells (n=5 mice/ treatment) for conditions in (B). Data represents mean ± s.d of 5 mice/condition with n=500 crypts per mouse measured (***, p=0.001, one-way ANOVA).

Supplementary Fig. 3

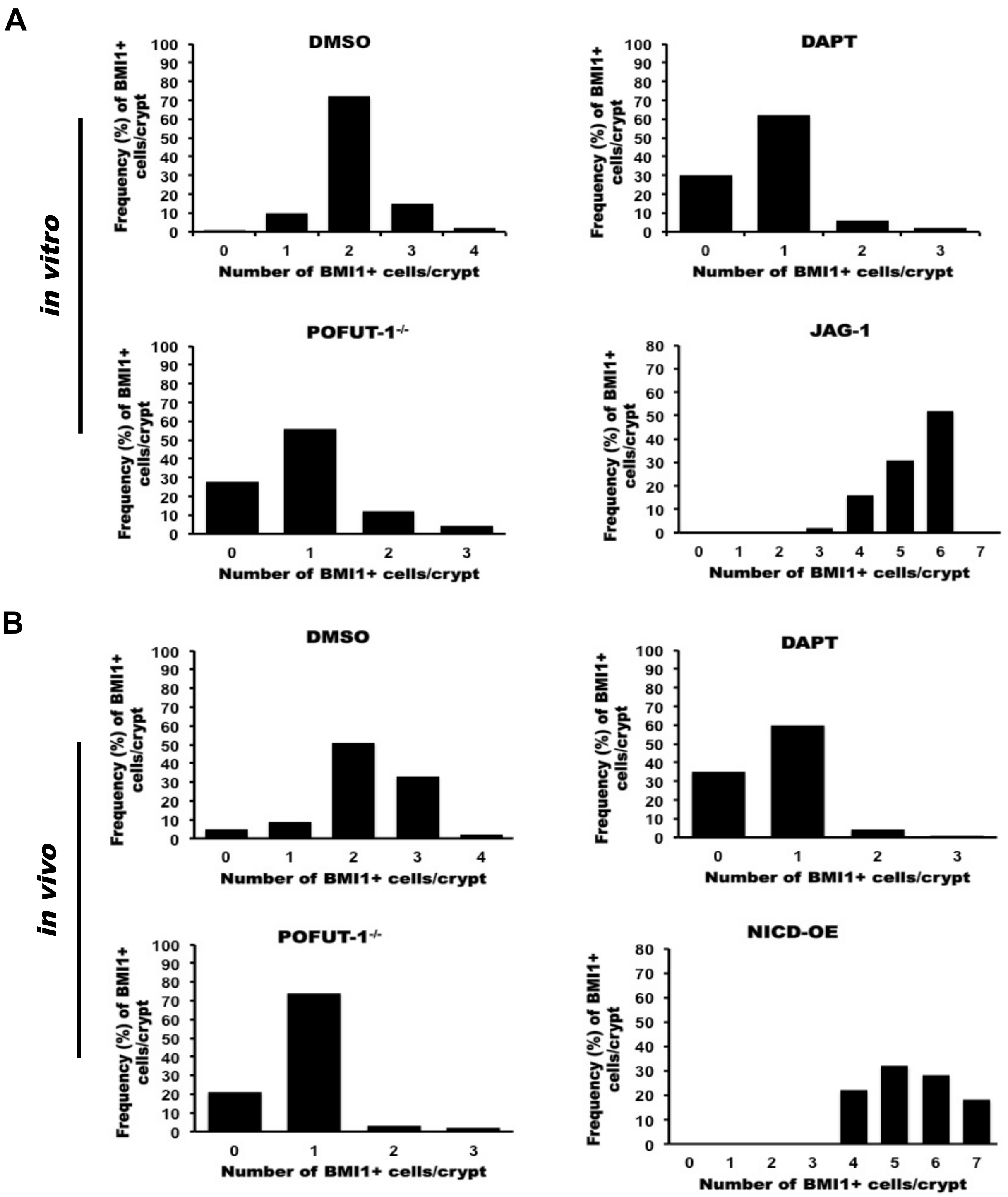

Supplementary Fig. 3. BMI1 expression in mouse organoids and intestinal crypts .

(**A**) Representative frequency of BMI1+ cells/crypt from *in vitro* assays (Fig. 1a, Fig. 1b) from murine LGR5-EGFP ISC organoids treated with DMSO, DAPT or JAG-1, and LGR5-EGFP-creER/ POFUT-1$^{\mathbf{flox/flox}}$ ISC organoids following 4-OH Tamoxifen induction of POFUT-1$^{-/-}$ phenotype. The data represent n = 500 organoids/condition. (**B**) Representative frequency of BMI1+ cells/crypt from *in vivo* assays over the entire small intestine (Fig. 2a, Fig. 2b) from LGR5-EGFP mice treated with DMSO or DAPT, Tamoxifen-induced LGR5-EGFP-creER/POFUT-1$^{-/-}$ mice, and Tamoxifen-induced LGR5-EGFP-CreERT2/Rosa26-YFP-NICD mice. The data represent n = 500 crypts/condition.

Supplementary Fig. 4

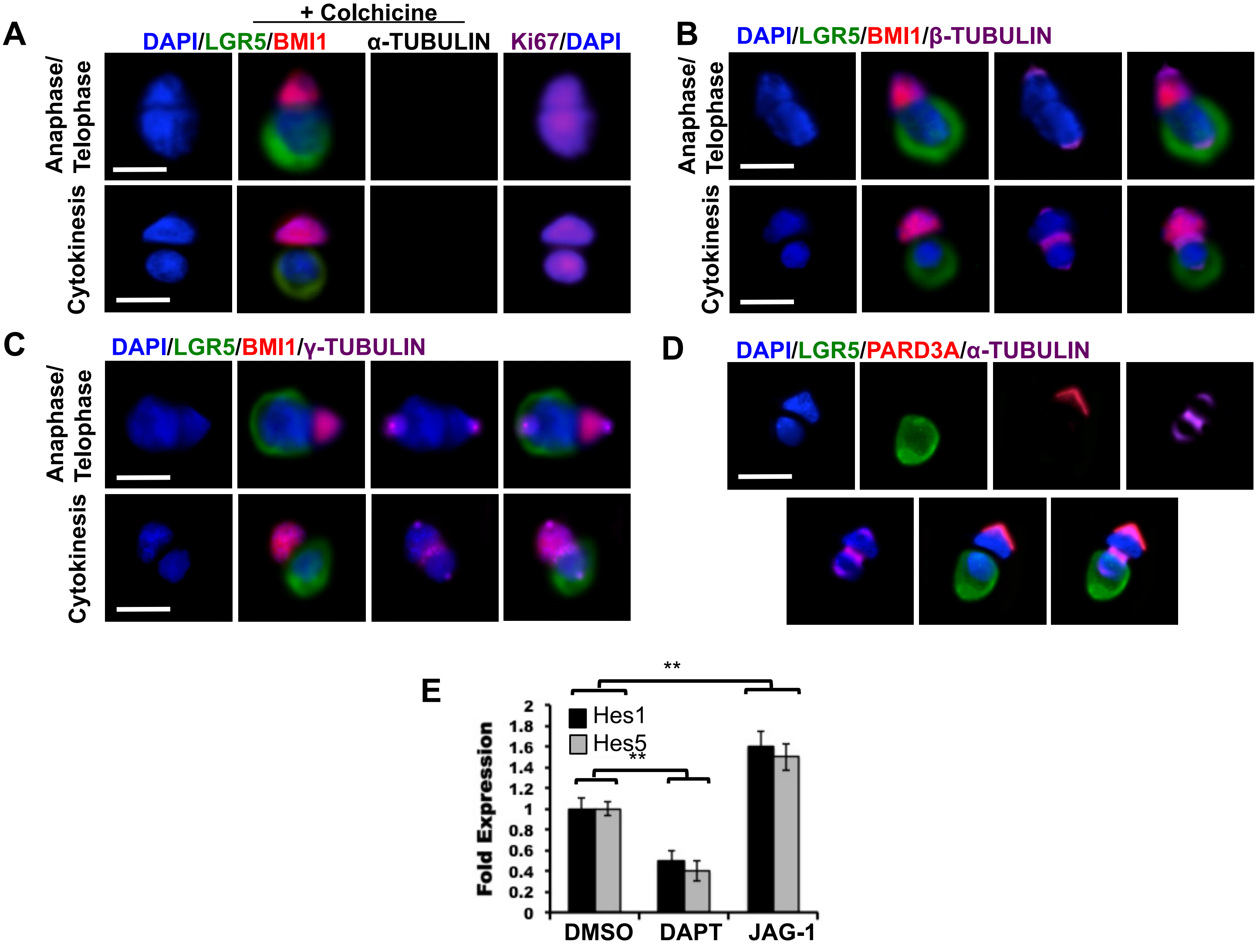

Supplementary Fig. 4. Asymmetric division in ISC organoids Related to Fig. 3.

(A) Representative co-IF of a LGR5-EGFP ISC daughter pair during anaphase/telophase (top) and cytokinesis (bottom) following 4 hour treatment with microtubule depolymerization agent (Colchicine). IF staining: Anti-GFP detects LGR5 (green), BMI1 (red), Ki67 (purple). DAPI (blue) labels nuclei and scale bar represents 50µm. **(B)** Representative co-IF of a LGR5-EGFP ISC daughter pair during anaphase/telophase (top) and cytokinesis (bottom). IF staining: Anti-GFP detects LGR5 (green), BMI1 (red), and β-TUBULIN (purple). DAPI (blue) labels nuclei and scale bar represents 50µm. **(C)** Representative co-IF of a LGR5-EGFP ISC daughter pair during anaphase/telophase (top) and cytokinesis (bottom). IF staining: Anti-GFP detects LGR5 (green), BMI1 (red), and γ-TUBULIN (purple). DAPI (blue) labels nuclei and scale bar represents 50µm. (**D**) Representative co-IF of a LGR5-EGFP ISC daughter pair just prior to completion of cell division. IF staining: Anti-GFP detects LGR5 (green), asymmetric cell fate marker PARD3A (red), and α-TUBULIN (purple). DAPI (blue) labels nuclei and scale bar represents 50µm. (**E**) Single LGR5-EGFP ISCs were treated with DMSO (control), 10uM DAPT or 1uM JAG-1 for 16h in a pair cell assay (Fig. 3b). Shown is RT-PCR data for expression of NOTCH effector genes Hes1 and Hes5 under each experimental condition (performed in triplicate and presented mean ± s.d..; $p = 0.01$; Student t-test for statistical significance).

Supplementary Fig. 5

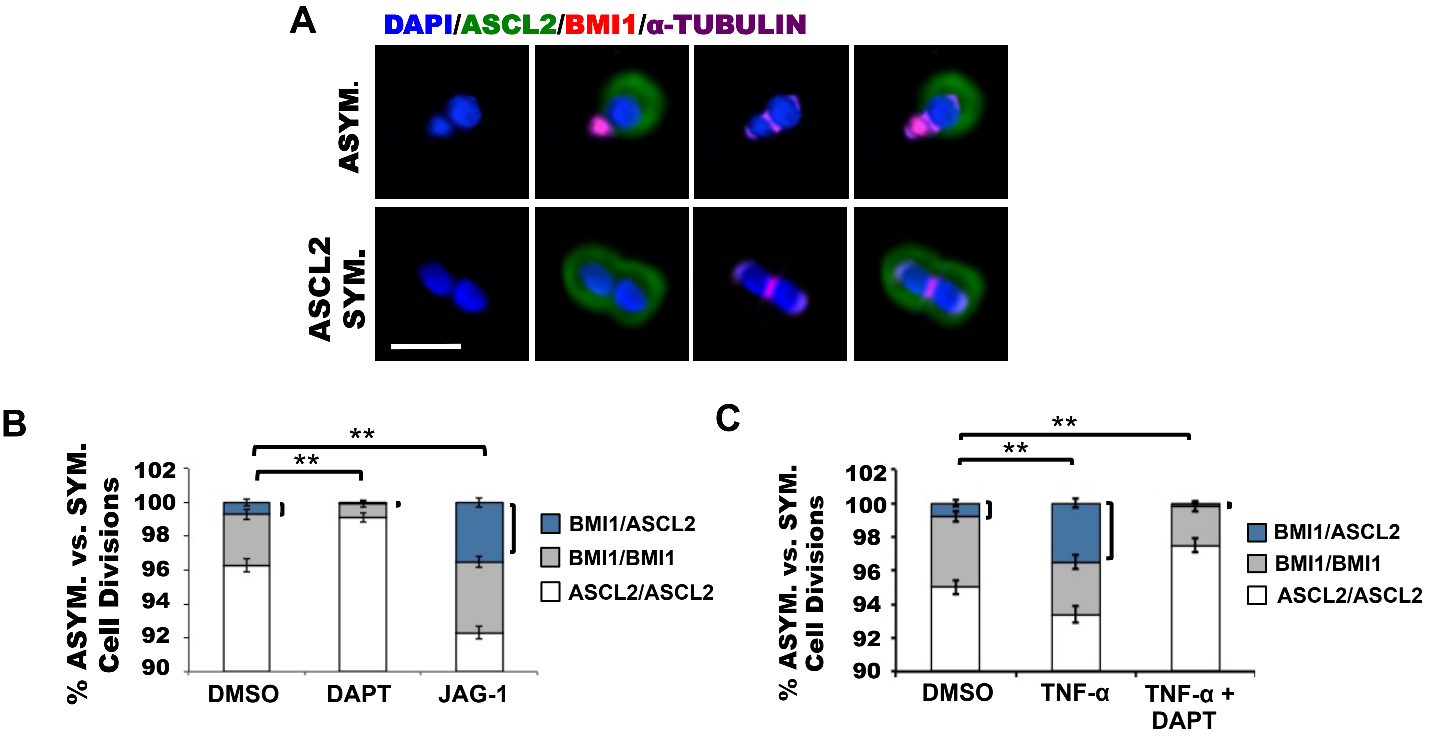

Supplementary Fig. 5. BMI1+/ASCL2+ asymmetric division *in vitro*.

(A) ISC daughter pair just prior to completion of asymmetric (top) or ASCL2+ symmetric (bottom) cell division. ASCL2 (green), BMI1 (red), α-TUBULIN (purple), DAPI (blue). Scale bar: 50µm. (**B**) LGR5-EGFP ISCs were treated with DMSO, 10uM DAPT or 1uM JAG-1 for 16h in a pair cell assay. Shown is percentage of ISCs undergoing BMI1+/ASCL2+ asymmetric (blue), BMI1+/BMI1+ symmetric (grey), or ASCL2+/ASCL2+ symmetric (white) cell division determined by co-IF for ASCL2, BMI1, and α-TUBULIN expression (**, p = 0.004, one-way ANOVA). Data represents mean ± s.d from three independent experiments with n = 500 daughter pairs/replicate. **(C)** LGR5-EGFP ISCs were treated with 10ng/ml TNF-α for 72 hours with simultaneous administration of DMSO or 10uM DAPT during the last 48 hours. Shown is percentage of ISCs undergoing BMI1+/ ASCL2+ asymmetric (blue), BMI1+/BMI1+ symmetric (grey), or ASCL2+/ASCL2+ symmetric (white) cell division (**, p = 0.002; one-way ANOVA). Data represents mean ± s.d from three independent experiments with n = 500 daughter pairs/replicate.

Supplementary Fig. 6

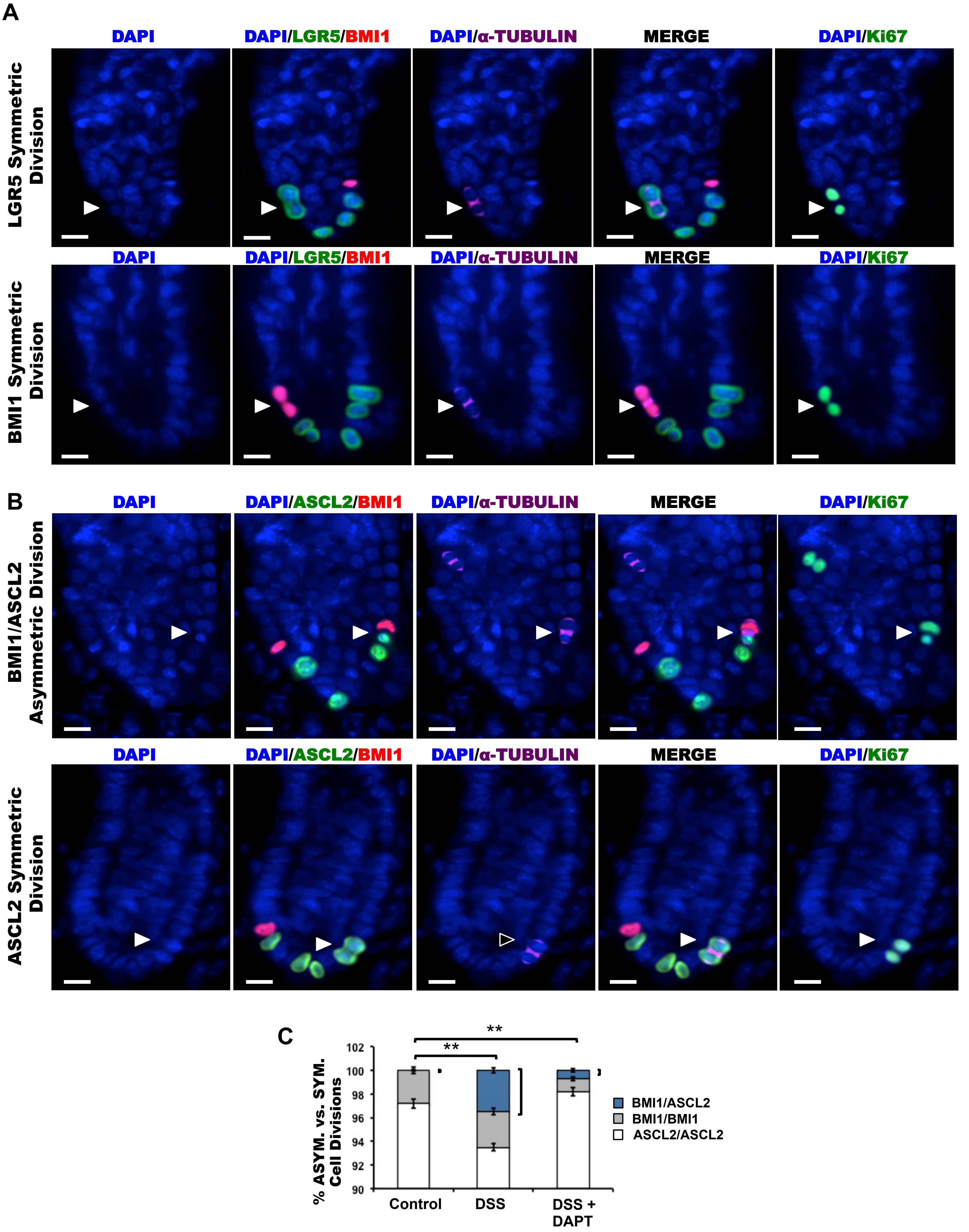

Supplementary Fig. 6. Murine *in vivo* ISC division. Related to Fig. 4.

(A) LGR5-EGFP mice were administered DMSO by i.p injection. Shown are representative co-IF images of intestinal crypts from the duodenum demonstrating LGR5+ (top panel) or BMI1+ (bottom panel) symmetric division. Anti-GFP antibody (green) detects LGR5-GFP+ cells; BMI1 (red), α-TUBULIN (purple) and DAPI (blue) labels nuclei. Ki67 staining (green) is also included. Scale bar represents 20μm. **(B)** LGR5-EGFP mice were administered 3% DSS in the drinking water for 5 days, followed by plain water for 5 days. During the last three days of the plain water diet, mice were injected i.p. with DMSO or DAPT every 12 hours. Shown are intestinal crypts from the duodenum demonstrating BMI1+/ASCL2+ asymmetric division (top) or ASCL2+ symmetric division (bottom). Co-IF: ASCL2 (green), BMI1 (red), α-TUBULIN (purple), Ki67 (green). Scale bar: 20μm. **(C)** Quantitative analysis from assay described in (B). Shown is the frequency of BMI1+/ASCL2+ asymmetric (blue), BMI1+/BMI1+ symmetric (grey), or ASCL2+/ASCL2+ symmetric (white) cell division. Data represents mean ± s.d. of 5 mice/condition with n = 500 TUBULIN+ dividing pairs/ mouse measured (**, p = 0.01; one-way ANOVA).
